# Peer Feedback on Collaborative Learning Activities in Veterinary Education

**DOI:** 10.3390/vetsci5040090

**Published:** 2018-10-17

**Authors:** Laura M. Dooley, Nicholas J. Bamford

**Affiliations:** Melbourne Veterinary School, Faculty of Veterinary and Agricultural Sciences, The University of Melbourne, Parkville, VIC 3010, Australia; n.bamford@unimelb.edu.au

**Keywords:** collaborative learning, communication, feedback, group work, veterinary education

## Abstract

Collaborative learning activities are an increasingly prominent feature of veterinary curricula that have been redesigned to achieve competency-based graduate learning outcomes. This evolution challenges the traditional individualistic approach to veterinary education and necessitates revisions to assessment and feedback practices to ensure constructive alignment. Peer feedback has been widely reported in the medical education literature as a teaching intervention in collaborative learning settings, with learning gains reported for students who receive and provide peer feedback. In this setting, peer feedback has been demonstrated to provide valuable formative feedback on professional behaviors and skills. However, there are very few such reports in the veterinary education literature to date. Barriers to the introduction of this approach can include teacher and student perceptions, and concerns around validity and reliability. This review aimed to provide an overview of current evidence regarding peer feedback on collaborative learning activities in higher education, and to explore opportunities and challenges for the introduction of peer feedback in the context of veterinary education. We contend that early and repeated provision of formative peer feedback can provide an opportunity to scaffold the development of crucial core competencies within veterinary education, including the self-regulated learning skills required to work in collaborative teams, and interpret and act on feedback.

## 1. Introduction

The recent paradigm shift in veterinary education towards competency-based outcomes [1,2] has resulted in fundamental redesigns of veterinary curricula globally [3]. An increasing focus on ‘non-technical’ or ‘professional’ competencies, including communication, team work, and a host of interpersonal skills, has seen the widespread introduction of collaborative work within these curricular features [4]. Collaborative learning pedagogies provide students with experiential opportunities to discover new information, apply prior knowledge, and solve problems, all while engaging in dialogue with peers. The ability to function within a team and to ‘*solicit, respect and integrate*’ contributions from others are considered core competencies of graduating veterinary students [2]. Therefore, there exists an imperative that veterinary educators provide opportunities to scaffold the development of these important transferrable professional skills within veterinary programs.

Despite the common use of collaborative and peer-based learning strategies, there remains limited evidence of effective assessment and feedback practices in the veterinary context [5]. The North American Veterinary Medical Education Consortium report identified collaboration as one of seven key professional competencies for veterinarians, including the ability to ‘*perform both peer- and self-assessment and discuss the strengths and weaknesses of collaboration*’ [1]. This competency reflects professional expectations that veterinarians provide feedback on the performance of their peers and are able to receive and respond appropriately to feedback on their own work. A fundamental ability to ‘*work cooperatively and effectively in a multidisciplinary team environment, including consensus building and conflict resolution*’ was also described. This report suggested that, like models employed in medical education [6], peer assessment can be used to provide formative feedback on the development of collaborative skills, while acknowledging that evidence in the veterinary context remains scarce [1].

Collaborative learning environments challenge the traditional individualistic approach to teaching and learning in the professions. To ensure constructive alignment of veterinary curricula, assessment and feedback practices must be designed with learning activities and intended learning outcomes in mind [7]. Where the ability to work in a team is a stated learning objective, peer feedback offers an opportunity to explicitly address intended learning outcomes that relate to team processes. The purpose of this literature review was to provide veterinary educators with an overview of the rationale for peer feedback in higher education and to discuss current evidence regarding the use of peer feedback in collaborative learning settings, particularly within medical education. We hypothesized that evidence within the veterinary education literature would be relatively lacking and sought to identify opportunities for future research. The intended focus was on reciprocal peer learning, in which students of equal status work collaboratively on tasks without delineation of specific teaching and learning roles, rather than pedagogies such as peer-assisted learning, in which students take on defined teacher or learner roles [8].

## 2. Literature Review

### 2.1. Peer Feedback in Higher Education

Peer assessment and feedback practices have been widely described in the higher education literature over several decades [9,10,11,12,13]. The ‘*arrangement for learners to consider and specify the level, value, or quality of a product or performance of other equal-status learners*’ [14] can be summative (quantitative) or formative (qualitative). Although definitions do vary in the literature, peer assessment most commonly refers to summative assessment activities, whereas peer feedback most commonly refers to formative assessment activities. Formative reciprocal peer feedback has been described as a ‘*communication process through which learners enter into dialogues related to performance and standards*’ [12], and this definition will be used in this review. The use of peer assessment in which formal scores or rankings are assigned might inhibit cooperation and promote negative learning practices [15]. Formative approaches, however, are typically met with less resistance from students [16,17]. Peer feedback may be provided on the products of learning activities (e.g., written pieces or oral presentations), the performance of specific tasks (e.g., demonstration of a clinical skill), or the processes involved in collaborative learning [9,15]. The engagement of learners with this teaching practice requires active co-construction of knowledge and practice standards, which has evolved from the educational theories of active learning [18] and social constructivism [19].

Collaborative learning activities provide a clear opportunity to introduce peer feedback. Indeed, the process of peer feedback itself has been described as a form of collaborative learning [20]. Effective feedback can assist students to understand or engage with knowledge or skills, and to develop the processing abilities required for future learning opportunities [21,22]. To achieve optimal student learning outcomes, feedback must be timely, clear, and of sufficient detail [23]. It should also be appropriately directed towards task outcomes, task processing, or self-regulated learning strategies, rather than towards learner characteristics which cannot be controlled by the individual [21,24]. Formative feedback specifically aims to improve performance and accelerate learning [25]. It therefore prioritizes student learning rather than the attainment of assessment goals, and strives to provide meaningful and actionable information about performance which can be utilized by learners in future tasks. For this reason, it has been suggested that formative peer feedback has a greater potential to impact student learning than summative peer assessment [12].

Formative peer feedback enables learners to take an active role in their ongoing development through engagement with self-regulated learning strategies. Self-regulation has been defined as ‘*self-generated thoughts, feelings, and actions that are planned and cyclically adapted to the attainment of personal goals*’ [26]. This manifests in learners who actively engage in recursive goal setting and monitoring activities through their learning experiences. Feedback is a critical enabler of self-regulated learning as it provides an external stimulus for the learner to review or monitor their own internal self-assessment of competency [15], and to make adaptive changes towards their learning goals [27]. Nicol and Macfarlane-Dick [28] propose a model of feedback practice in which students occupy a central role in regulating their goals and performance, while constructing their own understanding of feedback from external sources. They encourage teacher and peer dialogue around learning, rather than a one-way transmission of feedback, and suggest that peer discussion and feedback can expose students to alternative perspectives on goal attainment. Importantly, they also highlight that through engaging in peer feedback, students gain experience in evaluative processes, which can then be applied to the assessment of their own work. Additionally, there exists an emerging body of research examining socially shared metacognitive regulation that occurs in collaborative student groups [29,30,31,32]. This research provides evidence that in addition to self-regulation of individual learners, co-regulation of metacognition occurs within groups. In this way, peer feedback can contribute to the development of learners capable of self and shared regulation of metacognition; important skills for life-long professional learning.

### 2.2. Rationale for Peer Feedback

Peer feedback provides an opportunity to expand and diversify the delivery of capacity-building information to learners. There is a substantial body of evidence demonstrating learning gains for students both receiving and providing peer feedback [14,33,34,35]. Engaging in peer feedback can increase student motivation by fostering a greater sense of accountability and ownership of learning, leading to an increased time spent on task [12,34,36,37]. It provides an opportunity for earlier identification of errors or knowledge gaps [14], and encourages early reflection on progress and greater self-awareness [12]. Deep engagement with assessment criteria, exposure to different processes, and the internalization of standards are benefits for the peer assessor [14,34].

The requirement to deliver educational outcomes within increasingly resource-constrained higher education settings is a major consideration for the implementation of peer feedback. Students working in small groups have the capacity to provide immediate and individualized feedback within large cohorts. This may enable earlier identification of misconceptions or learning gaps. In the context of low-stakes formative feedback, peers can also provide more frequent and detailed feedback to inform ongoing skill development than it is possible for educators to deliver at scale.

The attainment of learning goals through engagement with collaborative learning tasks can be compromised due to social loafing (lack of contribution from individual students), freeloading (presenting the work of others as one’s own), or social and interpersonal difficulties [38,39,40,41]. For example, Hall et al. found that almost half of the students in a veterinary dissection class identified at least one peer who did not contribute productively to their group [38]. Therefore, if learning to work constructively within a team is a central learning objective of an activity, educators must move beyond a simple strategy of assessing task outcomes alone. While teachers are best placed to evaluate learning outcomes, in self-directed collaborative learning tasks, it can be argued that peers are best placed to evaluate team processes.

A drawback of peer feedback is that it is not provided by a domain or competency expert, as compared with teacher-driven feedback. This can result in variable accuracy of peer feedback [13], and reluctance to accept and act on feedback received [42,43]. However, there is evidence that differences in the nature of peer feedback can fundamentally change the manner in which the receiver uses this information, often leading to deeper review and revision, when compared to teacher feedback [44,45]. Yang et al. found that while teacher feedback was more likely to be adopted and lead to higher quality final outputs, peer feedback led to greater self-efficacy and improved learning processes [46]. It has therefore been argued that despite the risk of lower accuracy, the learning gains inherent in giving and receiving feedback, and the opportunity for increased speed and frequency of feedback, overall provide an acceptable trade-off [14,24,47].

### 2.3. Peer Feedback in Medical Education

Graduate medical doctors are required to work in small and large teams to deliver high quality care, and the ability to evaluate peers and provide feedback is a recognized core competency of medical graduates [48]. In medical education, peer feedback has been utilized in the context of pre-clinical and clinical education, for both technical and non-technical (professional) skills [49,50,51,52]. A systematic review of the reliability and validity of peer assessment practices in medical education across 28 studies demonstrated a variety of different approaches employed by educators [53]. The studies analyzed often designed their own instrument for peer assessment, with data on reliability and validity frequently lacking and many providing insufficient psychometric analysis. The authors argued that unvalidated or unreliable instruments should be avoided, and that further research is required to evaluate these tools, particularly with respect to their psychometric qualities. They also concluded that the most appropriate design of peer assessment and feedback activities is highly dependent on the specific educational context and the intended learning outcomes of the task. This highlights a need for well-designed research studies that report detailed evaluations of validated peer assessment instruments across a wide range of medical education contexts.

A context in which peer feedback has been extensively reported in the medical education literature is within problem-based learning (PBL) settings [54,55,56,57,58,59]. Papinczak et al. investigated the correlations between peer, self, and tutor assessments in a first year PBL setting, finding that self-assessment was less accurate than peer assessment in evaluating student performance [56]. Interestingly, this study also found that the correlations between peer and tutor evaluations improved over time as students gained more experience in peer assessment, providing evidence that this skill can be developed over time. A positive influence of formative feedback provided by peers in the development of cognitive, collaborative, and motivational attributes during PBL classes has been demonstrated, with particular gains observed in students with below-average initial performance, in part due to increasing the students’ sense of belonging within their group [58,59]. The benefits of peer feedback in PBL also extend to the students who evaluate others, with evidence that students undertaking evaluation of the professional behaviors of their peers also demonstrated improved scores for their own professional behaviors [57]. Taken together, these studies support the use of formative peer feedback within PBL settings in medical education, particularly for the evaluation of professional skills and behaviors.

Several recent reports in the medical education literature have provided evidence that peer feedback outcomes may assist in the identification of students at risk of later professionalism concerns during senior clinical training [60,61,62]. Two retrospective studies [61,62] demonstrated an association between poor peer feedback on collaborative tasks in pre-clinical settings and later professionalism lapses during clinical training. These findings support the notion that peer feedback in pre-clinical settings can be used to provide feedback on professionalism during early educational experiences, which is relevant to later performance in clinical settings. Further to the potential educational value to learners themselves, peer feedback could also be used by medical educators to enable targeted intervention strategies to address poor professionalism in individual students. Peer feedback can therefore form part of a proactive strategy to support and develop professional skills during the pre-clinical phase of education.

### 2.4. Peer Feedback in Veterinary Education

Professional competencies including communication, teamwork, respect for others, and awareness of personal limitations have been identified among the most important skills expected of veterinary graduates [63,64,65,66]. However, new-graduate veterinarians often lack confidence or are deemed to be deficient in these skills by employers [66]. Proficiency in a range of professional competencies is expected during the clinical year(s) of veterinary training, yet education and assessment practices regarding these skills during the early years of veterinary school often lack formality [67]. Collaborative learning activities are well placed to deliver these learning opportunities to pre-clinical students [68], and the introduction of such activities has now been widely adopted in veterinary curricula [3]. Despite this, it has been argued that the assessment of professional competencies in veterinary curricula is ‘*often sporadic or inconsistent*’ [5]. In collaborative learning environments, assessment can prove a particular challenge, as much of the learning process may not be directly observed by educators.

Our search of the veterinary education literature utilized Scopus, PubMed, and Web of Science databases using the operators ‘AND’ and ‘OR’ with a number of free terms pertaining to the topic in question, including ‘peer’, ‘self’, ‘feedback’, ‘assessment’, ‘group’, ‘collaborative’, ‘veterinary’, and ‘education’. English language publications without restriction of publication date were then screened for relevance and quality in an iterative process. There were many reports of collaborative learning pedagogies in the veterinary education literature [3,69,70,71,72,73,74], but relatively few of these studies described the use of peer feedback as a tool for learning from these activities. A report of team-based learning in an undergraduate veterinary program described the inclusion of a peer review process with both formative and summative components, although the impact of peer review was not specifically investigated [71]. Two reports of veterinary problem-based learning [69,72] also mention the use of peer assessment but did not detail the nature or outcome of these evaluations. A recent survey of veterinary curricular change across 38 veterinary colleges indicated that nine colleges included ‘*self- or peer assessments, or portfolios*’ in their curricula [3]. Peer assessment and feedback activities might also be a more commonly employed educational tool within veterinary programs than suggested by the peer-reviewed literature.

A recent study by Channon et al. explored the factors which contributed to group success in veterinary pre-clinical collaborative learning activities [39]. This study found that final marks on group assessments did not provide a good indication of how well the group worked together or the engagement of individual students. A need therefore exists to assess group processing tasks, and the professional skills required to execute these effectively, separately from the output of a group activity. Further, an investigation into veterinary student learning processes in case-based learning identified that reciprocity of teaching-learning processes within student groups was a critical element of learning [70]. In a range of collaborative learning settings common to veterinary education (including team-based learning and case-based learning), educators are not nested within individual groups to observe collaborative function. Therefore, it can be argued that peers are best placed to provide reciprocal feedback on group processes and professionalism in these settings.

There are limited reports of peer review or feedback in non-collaborative veterinary education settings. Several authors have reported using peers to provide formative feedback on communication skills during simulated consultation settings [75,76,77], which was often supplemented by instructor feedback or self-reflection activities. Formative peer feedback has also been reported as part of the assessment strategy for written radiological reports [78], pathology case reports [79], and oral presentations about biochemistry topics [80]. In each of these examples, however, the feedback was provided on individual outputs, rather than on the process or outcome of collaborative learning tasks. This lack of evidence for the use of peer feedback on collaborative learning in the veterinary education setting contrasts strongly with that reported in the medical education literature.

## 3. General Discussion

In the health professions, graduates enter an increasingly complex and collaborative working environment, requiring engagement with peers in a process of lifelong learning and professional development. Both medical and veterinary curricula have significantly expanded their emphasis on collaborative learning to develop these critical competencies, which has created a need for evolution in assessment and feedback practices. Existing literature suggests that peer feedback activities can provide a tool to develop complex professional skills and interpersonal behaviors that can otherwise be difficult to explicitly address in teaching activities, and that higher education students have the capacity to receive and provide constructive peer feedback. Additionally, the skills gained through engagement in peer feedback activities are reported to contribute to the development of self-regulated learning skills and self-awareness, and this may confer an improved ability to engage in self-reflective practices [36,81]. Taken together, this evidence presents a convincing case for the inclusion of peer feedback activities in professional education contexts.

Despite mounting evidence for the effectiveness and relevance of peer feedback in similar educational contexts, our review of the veterinary education literature revealed relatively few published reports of this teaching intervention. Existing reports in veterinary education contexts mostly described the use of peer feedback on individual tasks within clinical contexts, and we found scarce published evidence of peer feedback embedded within the early stages of veterinary education. We did find a limited number of reports of peer feedback in veterinary collaborative settings; however, this aspect was not a focus of these reports and the impact of this teaching intervention was not explicitly evaluated. This might suggest that the approach has been more widely adopted than is evidenced in the literature. In contrast, there is much evidence describing the use of peer feedback in medical education, particularly within collaborative learning settings in pre-clinical contexts. We therefore observed a gap in the existing veterinary education literature. Although pre-clinical medical education contexts provide the closest model for comparison to similar-stage veterinary education, existing studies have indicated that optimal design of peer assessment activities is highly dependent on the specific educational context of the intervention [10,33]. Therefore, we argue that there is a clear need for further research that evaluates this approach within veterinary contexts.

There exist several barriers to the introduction of formative peer feedback practices, which may have contributed to delayed uptake within veterinary education. Many of our students have not been exposed to this teaching practice during their pre-veterinary education, which is often focused on highly individualistic, competitive assessment practices [82]. Educators too may be unfamiliar with this teaching approach and concerned about student perceptions and the impact this might have on their role in the classroom [12,14]. Educators might also be concerned about the lack of validated peer feedback instruments, and reluctant to trial a novel instrument with limited (or no) evidence of effectiveness in enhancing student learning. Social processes can influence feedback outcomes; for example, peer friendship structures, popularity, collusion, and other interpersonal power differentials [14,83], and this may contribute to educator reluctance to introduce such an intervention. It can be argued, however, that these social processes are also present within the working environments that veterinary graduates will enter, and introduction of peer feedback practices within the low-stakes environment of formative feedback in early veterinary education may help to equip students to better navigate interpersonal processes in their later careers.

For many students, the idea of directly evaluating their peers is uncomfortable and at odds with their traditional conceptualization of the role of peers in the educational experience. A number of authors have explored student perceptions of peer feedback and have described situations of initial anxiety related to the task [84,85,86,87]. However, most studies of student perceptions have been in the context of summative peer assessment, rather than purely formative feedback, and it has been suggested that resistance to formative peer feedback is less common than summative peer assessment [16]. Student anxiety related to formative peer feedback has been shown to decrease with time, and acceptance of this practice improves with experience [20,37,51]. This suggests that the delivery and receipt of peer feedback is a competency which can be developed over time. Therefore, early and repeated inclusion of this practice during a professional degree is likely to improve competency levels at the time of graduation.

Specific interventions to reduce student resistance, scaffold skill development, and improve engagement in peer feedback practices have been explored. The provision of training in assessment and feedback processes prior to the activity, and the use of standardized and transparent feedback forms, can reduce student anxiety and improve the quality of feedback provided [14,84,88,89]. The structure and training provided by educators to facilitate peer feedback process can be faded over time, resulting in scaffolding of skill development and increasing transfer of responsibility to the learner [90]. Additionally, fostering a culture that emphasizes safety around giving and receiving peer feedback can further reduce student apprehension [51,91,92]. Structured reflection and goal-setting activities following the receipt of peer feedback has been suggested to enhance the efficacy of this approach through the stimulation of self-regulated learning processes, leading to an iterative learning process [21,24,93]. Inclusion of these design elements may enhance the efficacy of this teaching intervention within veterinary education.

To achieve a complex professional competency, students often require repetition of experience and application across a variety of contexts [94]. These competencies should therefore be developed in a spiral manner across the curriculum, with students gaining a depth of experience as they progress and revisit the competency in different situations. Over time, the balance of structured support from educators can be altered to facilitate increasing independence of learners. In contrast to medical graduates, veterinary graduates are less likely to enter structured post-graduate training programs immediately upon graduation. Rather, veterinary graduates are likely to enter small private clinical practices with highly variable levels of supervision provided. New veterinary professionals therefore have an immediate need to engage in collaborative learning processes with colleagues and utilize self-evaluation of competence to monitor and adapt their veterinary professional knowledge to the workforce. Equally, to optimize the transition of new graduates to the profession, employers and colleagues are required to engage in dialogue regarding their performance, including structured or unstructured review and feedback practices. For these reasons, we highlight the importance of being able to provide and receive formative peer feedback for modern veterinary graduates.

## 4. Conclusions

Educators in the health professions are increasingly using peer feedback to facilitate the attainment of non-technical (professional) competencies. In contrast, the implementation of peer feedback as a formative approach to the assessment of collaborative learning activities in veterinary education appears to have lagged behind progress in medical education. There is evidence that student engagement and satisfaction with peer feedback practices improves with experience. Therefore, we contend that early and repeated inclusion of peer feedback approaches in veterinary curricula can provide an opportunity to scaffold the development of the crucial self-regulated learning skills required to work in collaborative teams and interpret and act on feedback. Initial implementation trials could be modelled on existing literature in medical education [54,55,56,57,58,59]. Any such interventions should be thoroughly evaluated and reported, so that veterinary educators can learn from the experience of others to optimize peer feedback processes within their learning contexts. As a profession that is entrusted with self-regulation, there is an imperative for veterinary educators to introduce structured learning pedagogies that enhance the ability of graduates to engage in peer review practices within collaborative professional environments.
